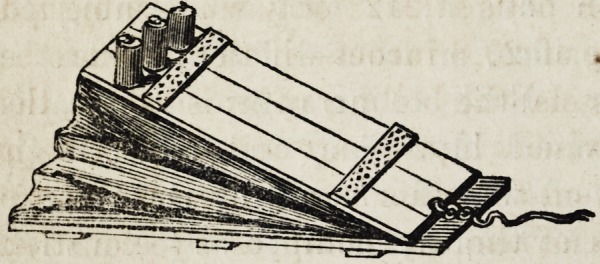# Hydrogen

**Published:** 1858-10

**Authors:** R. N. Wright


### ARTICLE II.

### *Hydrogen.* By Prof. R. N. WRIGHT, M. D.

*History.*—Cavendish was the first who accurately described this substance, about the year 1766—an account of which was given in the Philosophical Transactions, under the term inflammable air. Under the name of *phlogiston,* because supposed to constitute the essence or basis of flame, it had been confounded with many other inflammable gases. The term hydrogen was applied to it, because its oxidation rapidly produces water, a name, compounded of the Greek ϛδωρ, water, and γεννaω, I generate, or give rise to.

*Preparation.*—Nearly all the methods in common use for the preparation of hydrogen, consist directly or indirectly in the decomposition of water.

First. If the vapor of water is passed over bits of red hot iron in a porcelain tube or gun barrel, hydrogen will be obtained and quite pure. The following is the rationale of the process; a very strong affinity existing between the iron and the oxygen, the latter substance is appropriated by it, and oxide of iron produced, which remains in the tube, while hydrogen having nothing to combine with passes over, and may be collected in a bell glass, or other gas-holder, over the water of a pneumatic trough.

The annexed figure affords an illustration of the form of apparatus best adapted to this process.

A represents an ordinary plumbago, or fire-brick furnace, through the fire chamber of which passes the tube B, filled with small bits of iron, which must be heated to redness, (iron turnings or refuse machine card teeth answer the purpose best.) C, is a common glass retort, about half filled with water, which is made to boil by means of the spirit lamp D—placed beneath—the water boils, its vapor passes through the tube B, where it is decomposed—the oxygen uniting with the iron, while the hydrogen proceeds onward, and escaping by the delivery tube E, is passed through lime water contained in the wash bottle F, by which it is rendered more pure, and finally escaping through the tube G, is collected at the trough for experiment.

Second. A method greatly preferred to the above, and that indeed generally practiced, especially when large quantities of gas are to be obtained, is the decomposition of water by means of iron or zinc and sulphuric acid. If iron be used it is oxidized at the expense of the water, the sulphuric acid combining with the newly formed oxide, producing sulphate of iron, which being dissolved by the water, remains in solution, and from which it may be readily obtained, in the form of green crystals, by subsequent evaporation. Zinc is generally substituted for iron, and is on many accounts preferable; it should be first granulated, a process easily accomplished by melting the metal in an iron or other pot, and pouring it from a height (say standing on a common chair) into a tub of cold water. Sheet zinc however, where it can be obtained, is better even than this. In this case the decomposition is the same as in the other, but the results are somewhat different. Water is decomposed, its oxygen unites with the zinc forming oxide of zinc, with which the sulphuric acid combines, forming sulphate of zinc, which remains in solution in the vessel, and from which it may be obtained in white crystals by evaporation.

**NOTE.—The article on OXYGEN, published in the last number of the Journal, contains several typographical errors, for which the author would apologize by stating that it was published before he had opportunity of correcting the proof.**

DECOMPOSITION.

| Materials used. | Consist of | parts | Process completed. |
|---|---|---|---|
| 49 Diluted sulphuric acid. | Hydrogen, . . | 1 | 1 Hydrogen. |
| | Oxygen, . . . | 8 | |
| | Sulph. acid, | 40 | |
| 32.52 zinc. | zinc, | 32.52 | 80.52 Sulphate of oxide of zinc. |
| 81.52 | | 81.52 | 81.52 |

Expressed by symbols would read thus:

$HO + SO_3$ and $Zn = Zn\,O + SO_3, + H$, or if iron be used instead of zinc, thus:

$$HO + SO_3 \text{ and } Fe = Fe\,O + SO_3, + H.$$

The simplest form of apparatus which can be used for generating hydrogen with the above materials, is the following:

A represents a common wide mouthed green glass bottle—a pickle or preserve jar answers the purpose perfectly well—into the cork stopper of which is inserted a long glass funnel, B, reaching nearly to the bottom of the jar, for the purpose of supplying acid and water at pleasure, and a delivery tube, C, so curved as to communicate readily with the water of a pneumatic trough. The granulated zinc is introduced into the bottle in quantity sufficient to make all the gas which may be required at the time—so that it may not be necessary to open the apparatus during the process—having carefully inserted the cork, pour in the diluted acid, immediately brisk effervescence will occur, attended with the free and rapid evolution of hydrogen, pure or otherwise, according to the purity of the materials used.

There is another form of apparatus (which I would especially recommend to my readers) called by the inventor

"*Self Regulating Hydrogen Generator.*" It is extremely convenient and admirably adapted for the preparation of the gas, either on a large or small scale, and if perfectly tight may be kept in action for almost any length of time. The annexed cut represents the form in which it is generally constructed for table use. C is a large glass jar, about six inches wide, and twelve high, placed upon the wooden support, G—into this wooden stand, are securely fastened two iron or brass uprights, D D, which have, sliding freely upon them, two clamps, E E, which clamps sustain a bell glass, A, closed at the top with a brass cap and stop-cock, F, thus maintaining it at a proper height in the large vessel, C; in the bell glass A, is suspended by means of copper wires, a perforated copper cup B, on which the zinc in small pieces, is placed. When it is to be used, we proceed in the following manner: the zinc must be cut into fragments of the proper size, and introduced into the bell glass A, after which the whole apparatus may be arranged as represented in the figure—now pour the diluted acid into the large jar, until it is about two-thirds full, and open the stop-cock, upon which the fluid will attain the same level in both vessels; by this means the zinc will be immersed, and action will at once commence. When time has been allowed for the escape of the first portions, (which are always contaminated with atmospheric air,) close the stop-cock, and the gas will quickly begin to accumulate, which will continue until all the liquid has been expelled from A to C, thus leaving the zinc uncovered, action now ceases and will not recommence until by the escape of gas from the stop-cock, the acid is again brought into contact with the zinc, when action at once recommences, and continues until the bell glass is again filled with hydrogen. When it is desirable to prepare the gas on a large scale, a generator like the above, but made of sheet copper, is found to answer best.

*Properties.*—The peculiar odor which is perceived when hydrogen, as ordinarily made, is allowed to escape into the atmosphere, is not to be ascribed to any property possessed by the gas itself—but is plainly traceable to an oily combination of carbon and hydrogen, which is distinctly perceptible when hydrogen made by using iron as the deoxidizing agent, is passed through alcohol. The gas prepared in the ordinary way is never pure, always containing some sulphuretted hydrogen and carbonic acid, a difficulty which may to a considerable extent be remedied by passing it through some alkaline solution, as lime water, contained in the wash bottle.

Hydrogen is the lightest of all known substances, 100 cubic inches weighing only a little over two grains, (2.14) gr.; it is more than fourteen times lighter than atmospheric air, and sixteen times lighter than oxygen. For the above reason it is an excellent substance for the inflation of balloons.

Hydrogen stands perhaps, at the head of combustibles, in inflammability, taking fire with the greatest facility, and burning with a pale yellowish flame—the result of combustion, whether the gas be burned in air or oxygen gas, being water.

Though the flame of burning hydrogen emits so little light, the heat is very intense, as may be seen by holding many rather refractory substances in it, e. g., glass is readily melted, metallic wires heated to redness, and, if not very large, even melted.

A very beautiful illustration of the amount of heat produced during the combustion of hydrogen, is the following: pour iron filings in a steady stream from a small elevation, (12 or 18 inches,) upon a jet of ignited hydrogen, as soon as they come in contact with the flame, they become white hot, and, borne upon the heated current, dash in every direction, producing a fire tree, equal in beauty to some of the best fire-works.

If oxygen and hydrogen be mixed together in the proportions of one volume of the former to two of the latter, and a lighted match be applied, they will instantly unite with explosion, producing water ; the same will result if an electrical spark, or the galvanic current be passed through such a mixture. Though such is always the result if the gases are mixed in the above proportions, there will be no explosion if either be much in excess ; if hydrogen should greatly predominate, the mixture will take fire and burn quietly ; if, on the other hand, oxygen should be in large excess, the combustion of a burning body immersed in the mixture, will be rendered much more intense.

Hydrogen will not support combustion, as may be shown by inverting a bell glass containing it, over a lighted candle, the gas will burn quietly at the mouth of the jar, but the candle will be extinguished as soon as immersed in it.

One of the most remarkable of the phenomena connected with it, is the effect produced when it comes into contact with platinum, in a finely divided state ; but what is the absolute character of the changes which occur, remains still in obscurity. The following is a good mode of preparing the platinum: fasten some blotting paper in a small circular frame, having a few fine wires passing from side to side, drop upon it some solution of platinum, and hold it in the flame of a spirit lamp, until nothing remains but a consistent grey colored ash ; prepared in this way it is called spongy platinum, and instantly becomes heated to redness when exposed to a jet of hydrogen gas. This substance is used in the production of instantaneous light, in the once much used Döbereiner's lamp, which is nothing but a self-regulating hydrogen generator, such as we have described already, so contrived that when the stop-cock is opened, the jet will fall upon a piece of spongy platinum, which, becoming immediately red hot, kindles the gas. A is the outer glass vessel for the diluted acid, B, the small bell glass, which has suspended in it the zinc, C. D is a spring stop-cock, which, on being opened, allows a jet of gas to issue, and fall upon the ring of spongy platinum, E.

When hydrogen is generated in an ordinary gas bottle, having inserted in the cork a small glass tube, and ignited as it escapes from the jet, upon holding over it a glass tube 12 or 18 inches in length and about one inch in diameter, a loud, clear and distinct musical tone will be produced, which is explained by the following theory. The hydrogen ascending in the tube, mixes with the air above, producing thus explosive compounds, which explosive mixtures are formed and exploded in such rapid succession, that the ear is incapable of perceiving any interval between them, hence is produced an apparently continuous sound.

Hydrogen may be respired without injury for some time, the only unpleasant effects resulting from it being the production of a temporary alteration of the voice ; and though no animal could live more than a few seconds in an atmosphere of pure hydrogen, its death would be occasioned by the absence of oxygen, and not from any poisonous quality appertaining to the hydrogen.

In order to show satisfactorily, that water is produced whenever hydrogen is made to combine chemically with oxygen, it is only necessary to burn it in a portion of confined air, (e. g.) if an empty bottle be well cleansed and inverted over a jet of burning hydrogen, the inside will be quickly covered with a film of moisture which will gradually increase in quantity until it will flow in large drops. Putting together two measures of hydrogen and one of oxygen, and firing them by the electric spark, or lighted taper, they will combine, forming water; again, if into a suitable vessel some water (slightly acidulated) be poured, and a current of galvanic electricity passed through it, decomposition will result, hydrogen being disengaged at one pole of the battery, and oxygen at the other, and if the poles be previously covered with two small graduated bell glasses, (filled with water and inverted over them,) the resulting gases may be preserved for examination, when they will be found to bear exactly the relations specified above, viz. one volume of oxygen to two hydrogen.

The best mode of exhibiting the intense heat given out during the combustion of hydrogen, is to occasion its combustion in contact with oxygen, as they are issuing together from the same orifice, or from different orifices in contact with each other. Such an arrangement is called oxy-hydrogen, or compound blow-pipe, and (though the credit is not always given) was discovered by Dr. Hare, of Philadelphia; it has been variously modified as to construction, by many subsequent experimenters, but the principle remains the same in all. Newman, Wollaston, Clake, Hemming, Marcet, Maughham, Daniell, and others, have introduced methods intended to render the combustion of these gases safe, and among them all, Mr. Hemming's seems to have been preferred; it consisted of a metallic cylinder, five or six inches in length, entirely filled with pieces of wire of the same length, firmly packed and secured by having a metallic rod inserted in the centre, and driven tightly in the space between the wires, this constitutes a series of tubes, which allow the mixed gases to pass freely to the jet, but which will not allow the regurgitation of the flame on account of their excellent conducting powers. There is another form of jet, originally introduced by Mr. Daniell, for the combustion of coal gas with oxygen, which I greatly prefer to any other, and would recommend as perfectly safe; the subjoined cut illustrates it. A represents a milled coupling, by which attachment can be made to the gasometer, or bag containing oxygen, and C a similar coupling for connection with the hydrogen receptacle—it will be seen by inspecting the figure, that the oxygen passes down the centre of the apparatus, in the direction A D, that the hydrogen enters in the direction C B, entirely surrounds the oxygen jet, and escapes at a common orifice D, at which point they are burned—the hydrogen being ignited, and the oxygen let on in quantity sufficient to consume it completely.

One of the most convenient arrangements for holding gas is the India rubber bag, which may be obtained easily in any of our large towns, it is filled and emptied by means of flexible tubing of the same material, and when in use, is placed between two boards, as represented in the annexed engraving, upon which about two hundred pounds are placed, pressure being increased to any desirable extent by simply increasing the number of weights.

### *Water.*

Symbol HO or H + O—equivalent 9.

There are two compounds formed by the union of hydrogen and oxygen, viz. the *protoxide* and *deut-oxide,* or *bin-oxide* of *hydrogen.*

The first of these (water) which we shall describe in this paragraph, is formed whenever hydrogen gas is burned in contact with atmospheric air, or oxygen gas, a fact which was first satisfactorily proved by Mr. Cavendish in the year 1781. He found, upon burning known quantities of gas in a dry glass vessel, and carefully preserving the residue, that water was formed in exact proportion to the weight of the gases consumed—a fact which has been repeatedly verified since by a great number of the most able and assiduous observers.

Ice. Sp. Grav. — 0.916.

*Properties.*

Water is colorless and transparent when pure, tasteless and inodorous; it freezes at 32° F., boils at 212° of the same scale, and although water when kept from all agitation, may be cooled below 32° without freezing, ice cannot have its temperature raised above 32° without being reconverted into water. Water boils at 212° only when subjected to a barometeric pressure of 29.8 inches—if the pressure be increased (as in close vessels) the boiling point is raised, the same thing happens if viscid liquids are mixed with it or salts dissolved in it; if, on the other hand, pressure be removed, either by means of the air pump or by elevation above the sea level, the boiling point of water is lowered, indeed by means of air pump exhaustion ebullition will go on briskly even when the thermometer indicates 72°. Water boils under ordinary circumstances at 212° because, at that temperature its vapor acquires elastic force enough to overcome the resistance of the air—at all lower temperatures it simply evaporates, a process which is at all times going on at the surface of the earth and by which the atmosphere is furnished with its moisture, condensing into fogs, clouds, rain, hail, snow, &c.

A certain amount of air is always found in water, imparting to it a sparkling appearance and lively taste, the latter being entirely lost when it has been boiled, owing to the entire expulsion of air by heat, in which condition it will absorb varying portions of many other gases and gaseous combinations. I find the following statement in Graham, p. 315: "Rain-water generally affords 2½ per cent. of its bulk of air, in which the proportion of oxygen is so high as

30 per cent., and in water from freshly melted snow 34.8 per cent., according to the observations of Gay Lussac and Humboldt, while the oxygen in atmospheric air does not exceed 21 per cent. Boussingault finds that the quantity of air retained by water, at an altitude of 6 or 8000 feet is reduced to one-third of its usual proportion. Hence it is that fishes cannot live in Alpine lakes, the air contained in the water not being in adequate quantity for their respiration. The following table exhibits the absorbability of different gases by water deprived of all its air by ebullition.

"100 cubic inches of water at 60° and bar. 30, absorb of

| | Dalton & Henry. | | Saussure. |
|---|---|---|---|
| Hydrosulphuric acid, | 100 | cub. in. | 253 |
| Carbonic acid, | 100 | " | 106 |
| Nitrous oxide, | 100 | " | 76 |
| Olefiant gas, | 12.5 | " | 15.3 |
| Oxygen, | 3.7 | " | 6.5 |
| Carbonic oxide, | 1.56 | " | 6.2 |
| Nitrogen, | 1.56 | " | 4.1 |
| Hydrogen, | 1.56 | " | 4.6 |

"The results of Saussure are probably nearest the truth for hydrosulphuric acid and nitrous oxide, but for the other gases, those of Dalton and Henry are most to be relied on."

Water in a chemical point of view is a substance of the greatest importance; it combines with a vast number of other substances, through a very extensive range of affinities, forming definite compounds, which are either called aqueous combinations or hydrates, and is by far the most general and indispensable solvent we possess.

Nearly all salts are dissolved by it to a greater or less extent, their solution being generally attended with fall of temperature to a point sometimes below the freezing point of water, the salts being again deposited either amorphously or crystalline, as evaporation removes water and diminishes its capacity to hold so much in solution as at first.

There is, in some instances, a singular suspension of crystallization, even from solutions made at the boiling temperature, the process however commencing and going on to completion, from very slight causes. Sulphate of soda is generally used as an illustration ; if we dissolve two pounds of the salt in one pound of boiling water, taking care to filter the solution while hot—we may set it aside in a flask and it will remain fluid for a considerable time, if slightly covered so as to keep out particles of dust floating in the atmosphere ; this may be done by tying a piece of bladder over the mouth of the glass or by pouring a few drops of oil on the surface. Violent shaking under these circumstances will, sometimes, not occasion crystallization, but if a stick, bit of glass or some other solid (best of all a crystal of the same salt) be dropped into the solution, crystallization at once commences and proceeds with rapidity until the whole mass becomes solid with the exception of a few drops of fluid which remain on the top. There are attending this process, two circumstances worthy of consideration—first, the solid has much less capacity for heat than the fluid, inasmuch as heat enough is given out at the moment of solidification to render the outside of the vessel quite warm to the hands. Second, there is a change in bulk, the solid really occupying more space than the liquid, this is shown by accurately marking the point at which the fluid stands before solidification and observing that the mass stands at a sensibly higher point after the completion of the process.

In general, heat increases the capacity of water for dissolving salts, but several instances occur especially with salt of lime, in which solubility does not seem to be materially increased in this manner.

*Purity of natural waters and artificial means of purifying them:*

Undoubtedly the purest natural waters are those of rain or melted snow. After rain has been falling some time, and the first portions have dissolved out most or all of the soluble impurities contained in the air through which it has fallen, we may consider it for all practical purposes, as almost absolutely pure, containing as it does, little or nothing but a small quantity of air; this is only true, however, when the rain water has been received into perfectly clean porcelain or glass vessels, if allowed to touch metallic vessels or to come in contact with the earth it is at once contaminated to a greater or less extent. Water from melted snow has a decided advantage over rain water, in the fact, that at the moment of freezing, there is a strong tendency to reject all foreign matters, in however minute quantity, that may have existed in the liquid. In this case also, great care should be taken to receive the falling snow upon perfectly clean surfaces, otherwise it may become contaminated by absorption of impurities.

The three principal methods of purifying water artificially are, *subsidence, filtration* and *distillation.* The first process is perfectly simple and easily practicable at any time; there is nothing to be done but draw the given quantity of water into a suitable vessel, and allow it to remain at perfect rest for a period of time longer or shorter, according to the quantity of water and the amount of impurity suspended in it. This method only removes *mechanically suspended impurities,* (which subside to the bottom,) but does not affect substances actually dissolved in the water. When the supenatent liquid is apparently clear, it may be carefully withdrawn by means of a siphon, or decanted from a stopcock placed in the side of the vessel some distance from the bottom, so as to prevent the agitation of the sediment.

The second process or *filtration,* is that most commonly practiced on a large scale; the beds of reservoirs are covered to the depth of several feet with alternate layers of sand, gravel and stones, through which the water slowly percolates, being finally discharged by means of a large pipe, the reservoir end of which is covered with a metallic strainer. On a smaller scale, filters may be made of porous earthenware, capable of holding several gallons—into which the water is poured, and after the lapse of a brief space it will have passed slowly through and be found comparatively free of impurities, except such as are actually in solution—those of course remain. The laboratory filter is nothing more than a piece of bibulous paper, folded and inserted in a glass funnel ; the liquid to be filtered is poured upon it and allowed to drain quietly off, leaving impurities upon the paper.

The only reliable method of obtaining pure water for the laboratory or the arts is distillation. Water is poured into a vessel which will resist the action of heat and the temperature raised to 212° F., at which point the vapor is capable of overcoming the resistance of the air ; this vapor is allowed to escape into a vessel kept constantly cool, where it is condensed into pure water, having left foreign substances of all kinds in the boiler. Great care should be taken that the apparatus be thoroughly cleansed whenever used, nor should it ever be used for any other purpose—and if the apparatus be made of copper or any other metal, the first portions distilled should be thrown away, as they will most probably contain portions of metallic salts or oxides in solution, thus (without the above precaution) contaminating the whole result.

Of all the sorts of water on the surface of the earth, spring and river waters are in general the purest—and the substances most commonly present in hard water are sulphates and carbonates of lime ; indeed, in almost all mineral waters sulphuric and carbonic acids are found in combination with other substances. In saline waters, the salts of magnesia, chloride of sodium and salts of lime are most frequently found.

*Sulphurous waters* contain *hydrosulphuric acid* in small quantity, and it is this which imparts to them their property of discoloring certain metals, as *lead, silver, &c.*, as well as their peculiarly disagreeable taste and odor.

*Chalybeate waters,* as they are called, contain iron, most commonly in combination with carbonic acid, but the quantity present is never large, rarely exceeding two grains to the pound, and generally less than one grain.

### *Binoxide of Hydrogen.*

$HO_2$ or $H + 2O$. Eg. 17.

*History.* This singular compound was discovered by Thenard, in the year 1818, and was called by him oxygenated water.

*Preparation.* It is obtained by the action of dilute acids upon *hydrated binoxide of barium.* Salts of baryta are formed by the combination of these acids with the *protoxide of barium,* and the equivalent of *oxygen,* thus liberated from the *binoxide,* does not escape but combines with the water of the former *hydrate,* by which combination *binoxide of hydrogen or oxygenated water* ($HO + O$) is produced, afterwards remaining in solution in the water.

Thenard proposes to dissolve the binoxide of barium in very dilute hydrochloric acid, getting rid of the baryta by sulphuric acid precipitation, and after several repetitions removes the *hydrochloric acid* by *sulphate of silver,* finally using baryta to carry off the sulphuric acid. A modification of this process has been proposed, much more direct; it is to decompose the *binoxide of barium* with *hydrofluoric acid,* instead of *hydrochloric*—there is a rapid formation of *fluoride of barium* and *binoxide of hydrogen,* which latter may be freed from the *fluoride* by subsequent filtration, and brought to the proper density—1.452,—by air-pump evaporation.

*Properties.* This substance in appearance resembles water, having no color, and a slightly metallic taste; its effect upon organic coloring matters is analogous to chlorine, bleaching them rapidly by mere contact; it is not so volatile as water and has its permanency increased by dilution with that liquid and some of the stronger acids.

Heated up to about 60° F. it decomposes, effervescing briskly from separation of oxygen, and if the temperature be suddenly raised to that of boiling water, oxygen is liberated so rapidly that explosion ensues.

*Uses.* On the use of this substance, Graham has the following paragraph—pp. 321 and 322.

"The *binoxide of hydrogen* is a substance which it is exceedingly desirable to possess, with the view of employing it in bleaching and for other purposes, as a powerful oxidating agent; but the expense and uncertainty of the process for preparing this compound have hitherto prevented any application of it in the arts, or even its occasional use as a chemical re-agent."

## Figures and Tables

**Figure f1:**
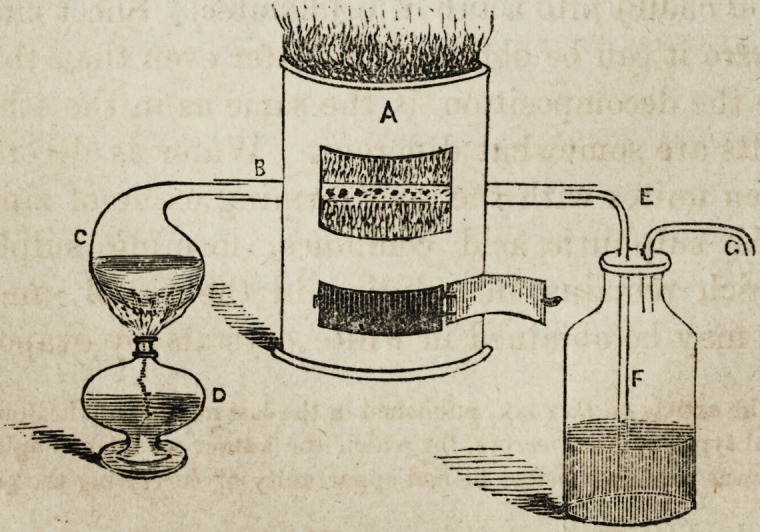


**Figure f2:**
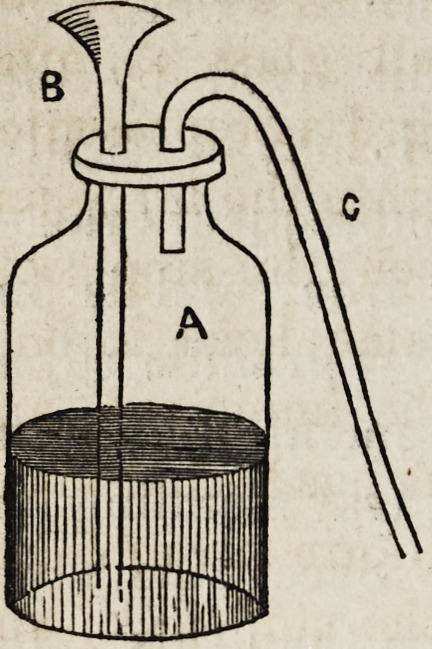


**Figure f3:**
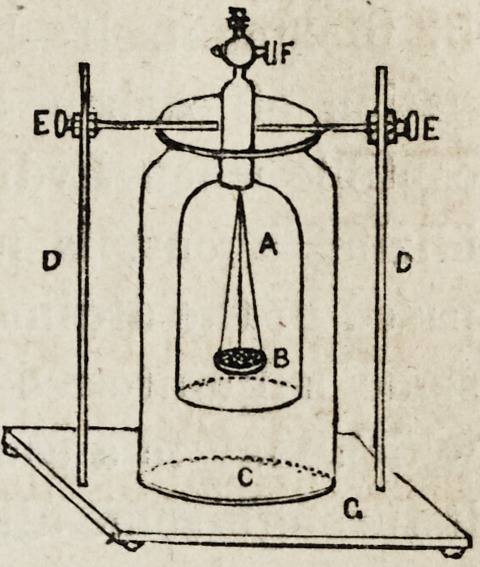


**Figure f4:**
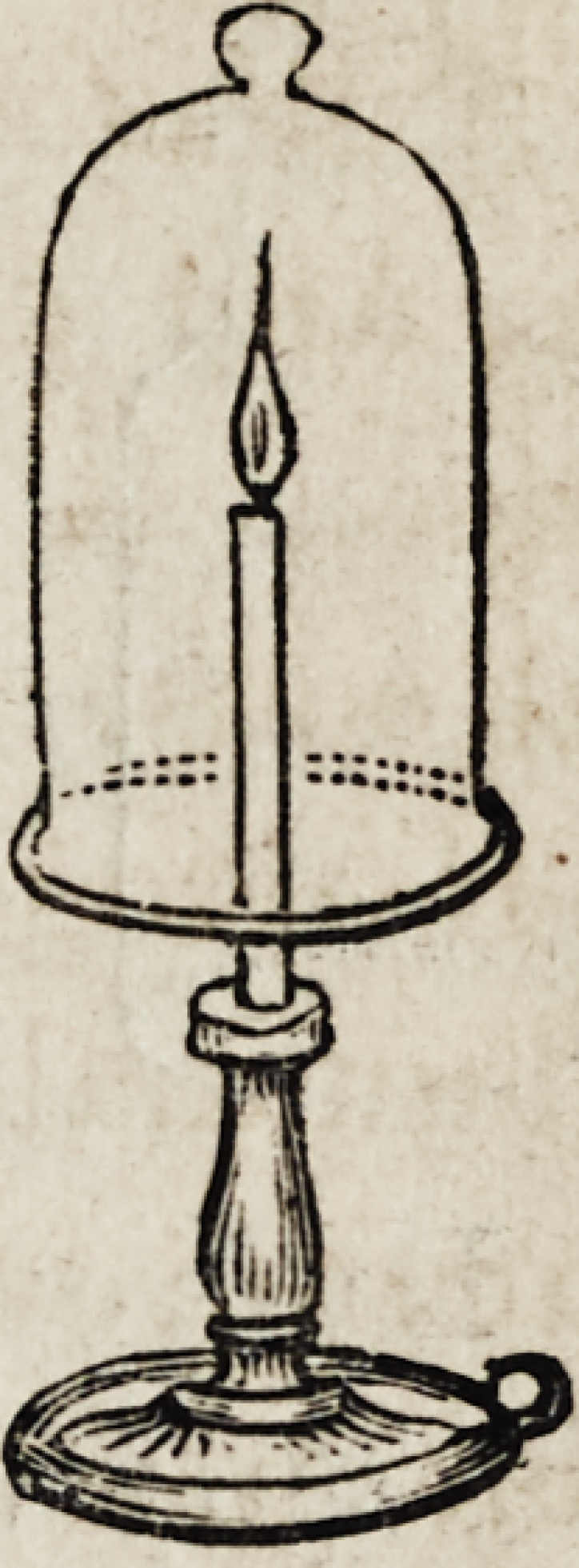


**Figure f5:**
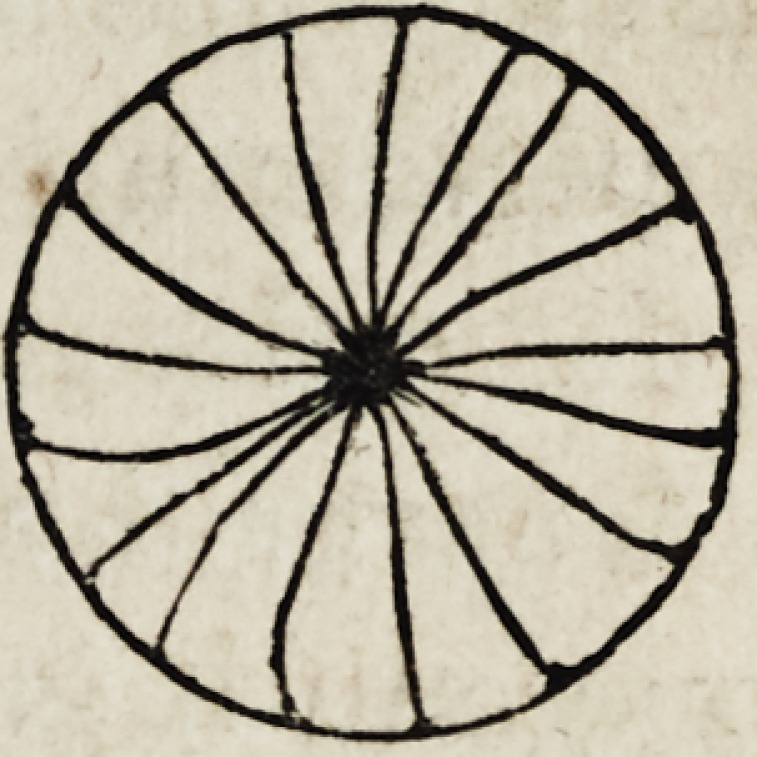


**Figure f6:**
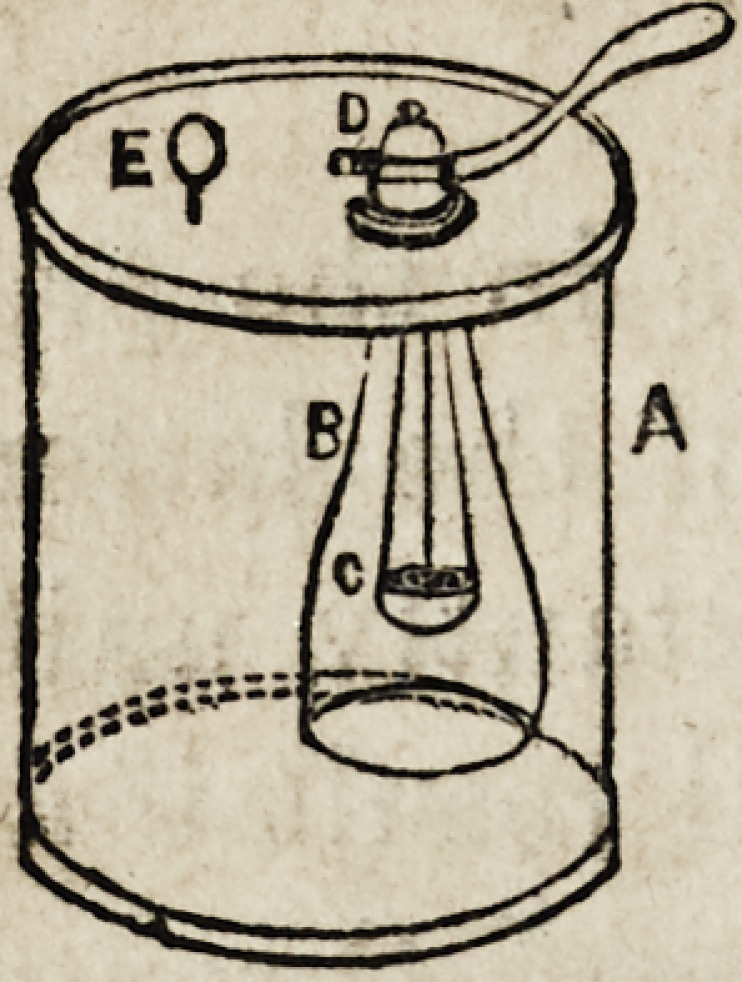


**Figure f7:**
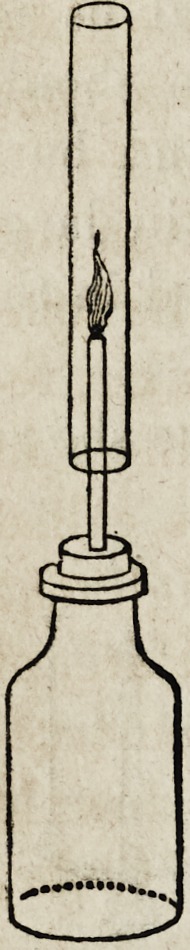


**Figure f8:**
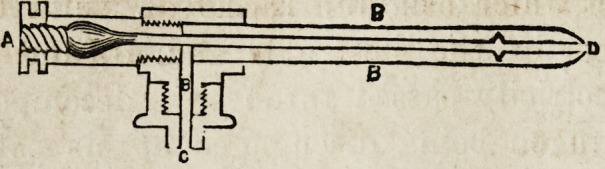


**Figure f9:**